# Autotrophic carbon fixation strategies used by nitrifying prokaryotes in freshwater lakes

**DOI:** 10.1093/femsec/fiy163

**Published:** 2018-08-18

**Authors:** Albin Alfreider, Victoria Grimus, Martin Luger, Anja Ekblad, Michaela M Salcher, Monika Summerer

**Affiliations:** 1 Institute of Ecology, University of Innsbruck, Technikerstraße 25, 6020 Innsbruck, Austria; 2 Institute for Water Ecology, Fisheries Biology and Lake Research, Federal Agency for Water Management, Scharfling 18, 5310 Mondsee, Austria; 3 Institute of Hydrobiology, Biology Centre CAS, Na Sádkách, 702/7370 05 České Budějovice, Czech Republic

**Keywords:** chemoautotrophs, nitrifiers, lakes, CO_2_ fixation pathways

## Abstract

Niche specialization of nitrifying prokaryotes is usually studied with tools targeting molecules involved in the oxidation of ammonia and nitrite. The ecological significance of diverse CO_2_ fixation strategies used by nitrifiers is, however, mostly unexplored. By analyzing autotrophy-related genes in combination with *amoA* marker genes based on droplet digitial PCR and CARD-FISH counts targeting rRNA, we quantified the distribution of nitrifiers in eight stratified lakes. Ammonia oxidizing (AO) *Thaumarchaeota* using the 3-hydroxypropionate/4-hydroxybutyrate pathway dominated deep and oligotrophic lakes, whereas *Nitrosomonas*-related taxa employing the Calvin cycle were important AO bacteria in smaller lakes. The occurrence of nitrite oxidizing *Nitrospira*, assimilating CO_2_ with the reductive TCA cycle, was strongly correlated with the distribution of *Thaumarchaeota*. Recently discovered complete ammonia-oxidizing bacteria (comammox) belonging to *Nitrospira* accounted only for a very small fraction of ammonia oxidizers (AOs) present at the study sites. Altogether, this study gives a first insight on how physicochemical characteristics in lakes are associated to the distribution of nitrifying prokaryotes with different CO_2_ fixation strategies. Our investigations also evaluate the suitability of functional genes associated with individual CO_2_ assimilation pathways to study niche preferences of different guilds of nitrifying microorganisms based on an autotrophic perspective.

## Teaser

CO_2_ fixation strategies used by nitrifiers were investigated in lakes, suggesting that *Thaumarchaeota* using the 3-hydroxypropionate/4-hydroxybutyrate pathway were dominant in deep and oligotrophic lakes, whereas bacteria employing the Calvin cycle were important in samples with an elevated nutrient status.

## INTRODUCTION

Nitrifiers are chemolithoautotrophs, which are defined by their ability to use reduced inorganic nitrogen compounds as an energy source and inorganic carbon to fulfill the carbon need. Until recently, the two-step nitrification process has been considered to be catalyzed by two separate groups: ammonia oxidizing (AO) organisms, which include ammonia-oxidizing archaea (AOA, Könneke *et al*. 2005; Hatzenpichler 2012), ammonia-oxidizing bacteria (AOB, Kowalchuk and Stephen 2001) and nitrite-oxidizing bacteria (NOB). However, the discovery of bacteria that catalyze complete nitrification (complete ammonia oxidizers; ‘comammox’), members of the genus *Nitrospira*, has fundamentally expanded our view of the nitrification process (Daims *et al*. 2015; van Kessel *et al*. 2015). The first and rate-limiting step in nitrification, the oxidation of ammonia to hydroxylamine, is catalyzed by the enzyme ammonia monooxygenase (AMO). As all known bacterial and archaeal ammonia oxidizers harbour AMO, the gene encoding the alpha subunit of this enzyme (*amoA*) has become a well-established functional marker to analyze the distribution, diversity and ultimately the niche preferences of AOA and AOB in the environment (e.g. Bouskill *et al*. 2012; Meinhardt *et al*. 2015).

On the other hand, the ecological importance of different carbon fixation strategies used by nitrifying prokaryotes has not been paid much attention. This is quite surprising, considering the diversity of biochemistries of CO_2_ assimilation pathways that also might influence the distribution of nitrifiers in the environment. To date, six carbon assimilation pathways are known, whereof three are used by nitrifying prokaryotes (Berg 2011). The Calvin-Benson-Bassham (CBB) cycle is present in different genera of NOB including *Nitrobacter, Nitrococcus, Nitrotoga* and *Nitrolancea* (Daims, Lücker and Wagner 2016) and generally found in AOB belonging to Proteobacteria (Badger and Bek 2008). The enzyme responsible for the actual fixation of CO_2_ in the CBB cycle, ribulose-1,5-bisphosphate carboxylase/oxygenase (RubisCO), occurs in different forms characterized by specific catalytic properties (Berg 2011). Most AOB possess form IA RubisCO. This enzyme is known for its poor catalytic affinity for CO_2_, which is in some AOB (e.g. *Nitrosomonas eutropha* C91) compensated by carbon concentrating mechanisms that support better growth at low CO_2_ concentrations (Stein *et al*. 2007; Badger and Bek 2008). Form IC RubisCO is present in several AOB affiliated with different *Nitrosospira* species and the Gammaproteobacterium *Nitrosococcus oceanii*. Several AOB, including *Nitrosomonas sp*. Is79 and *Nitrosomonas* sp. AL212, even encode two copies of the RubisCO operon in their genomes (Bollmann *et al*. 2013). However, there is a clear lack of environmental studies addressing the ecological adaptation and specific niches occupied by AOB using different forms of RubisCO. A variant of the 3-hydroxypropionate/4-hydroxybutyrate (HP/HB) cycle operates in AOA (Könneke *et al*. 2014). This pathway was described as the most energy efficient aerobic carbon fixation cycle, which fits in well with the adaptation of AOA to nutrient-limited conditions. For environmental investigations, molecular tools targeting genes coding for the key enzymes 4-hydroxybutyryl-CoA dehydratase and acetyl-CoA/propionyl-CoA carboxylase are highly specific instruments to explore the diversity of autotrophic *Thaumarchaeota* using the HP/HB cycle for CO_2_ fixation (e.g. Yakimov, La Cono and Denaro, 2009, 2011; Bergauer *et al*. 2013; Hu *et al*. 2013; La Cono *et al*. 2013; Tolar, King and Hollibaugh 2013; Alfreider *et al*. 2017). Nitrifying bacteria using the reductive citric acid cycle (rTCA) are found in members of the genus *Nitrospira*, which include NOB and comammox (Daims, Lücker and Wagner 2016). In marine systems, the rTCA cycle is employed by the nitrite oxidizing *Nitrospina* species (Pachiadaki *et al*. 2017). The rTCA cycle is a reversal of the oxidative citric acid cycle (Krebs cycle) and forms acetyl-CoA from two CO_2_ (Berg 2011). This carbon fixation strategy was originally known to occur in Epsilonproteobacteria and *Aquificae* in anaerobic and microaerobic environments, due to the oxygen sensitivity of key enzymes in the cycle (Hügler and Sievert 2011). However, for *Nitrospira* it has been shown that enzymatic adaptations strengthen the O_2_ robustness of the rTCA cycle that allows the pathway to function also in aerobic habitats (Berg 2011). In environmental samples, the rTCA cycle is usually detected by targeting genes coding for the alpha or beta subunit of the ATP citrate lyase and the alpha subunit of 2-oxoglutarate:ferredoxin oxidoreductase enzymes (Hügler and Sievert 2011; Kovaleva *et al*. 2011; Noguerola *et al*. 2015; Alfreider *et al*. 2017).

The goal of this research has been developed based on the results of a former study, where the diversity of sequences coding for selected key enzymes in the HP/HB, CBB and rTCA cycle in six lakes were analyzed (Alfreider *et al*. 2017). In that study, the authors demonstrated that a significant part of the sequences was related to nitrifiers, suggesting that nitrification is a major source of energy for chemoautotrophs in these lakes. Specifically, sequences affiliated with the genus *Nitrospira* and *Thaumarchaeota*, using the rTCA and HP/HB cycle respectively, were mostly found in deep lakes. RubisCO form IA genes, related to members of the *N. oligotropha* lineage (cluster 6A) have been detected in different lake types and depths. However, as the study of Alfreider *et al*. (2017) was mostly based on sequence analysis from selected samples, the abundance, distribution and consequently the ecological niche preferences of different guilds of nitrifiers remained unexplored. For the present work, different strategies were developed in order to quantify the distribution of three CO_2_ fixation pathways of AOA, AOB and NOB in a variety of eight stratified lakes of different sizes and environmental characteristics. These ecosystems are characterized by distinct and stable concentration gradients of oxygen and different redox states of nitrogen, thus allowing the investigation of nitrifying prokaryotes in the ecological framework of measurable habitat heterogeneity. In this respect, we expect a vertical niche separation of nitrifying Thaumarchaeota and bacteria with different energy and substrate requirements, which is also linked to their different strategies for CO_2_ fixation. Digital droplet PCR (ddPCR) with specifically designed primers were applied to target the most abundant clades of sequences affiliated with nitrifiers in lakes. These values were compared with ddPCR derived *amoA* gene abundances of AOB and AOA, which also includes the analysis of comammox-*Nitrospira* based on a recently developed qPCR assay (Pjevac *et al*. 2017). Furthermore, catalyzed reporter deposition fluorescence *in situ* hybridization (CARD-FISH) enabled the microscopic analysis based on the taxonomic affiliation of dominant nitrifiers.

## MATERIALS AND METHODS

### Field work and chemical analysis

Eight lakes (Attersee-ATT, Hallstättersee-HAL, Millstätter See-MIL, Traunsee-TRA, Faakersee-FAA, IrrseeI-RR, Mondsee-MON, Weißensee-WEI), all of them located in Austria, were chosen based on their different thermal and chemical stratification patterns. The geographical location and the key morphometric and limnological parameters of the lakes are shown in Table 1. Single samples from different depths, covering the entire water column, were sampled during the summer stratification season before autumn/winter turnover started (Table 1). Exception was lake WEI, where samples down to 50 m water depth were taken. Sampling and analysis of environmental and chemical parameters were done according to the requirements of the Water Framework Directive in Austria (GZÜV). Subsets from the same samples were used for molecular analyses, CARD-FISH counts and water chemistry. For all lakes, multi-parameter probes (YSI Yellow Springs Instruments, OH) were used to obtain vertical profiles of temperature, dissolved oxygen (DO), pH and conductivity. Major anions and cations were determined either by ion chromatography or standard wet chemical analysis following ISO standard manuals (ammonium-ISO 5071; nitrate, sulfate-ISO 10 304–1; phosphate-ISO 6878, alkalinity-ISO 9963-1).

**Table 1.** Main characteristics of the studied lakes. Lakes are sorted by increasing water volume.

Lake^a^	Location	Sampling date	Altitude (m a.s.l.)	Area (km^2^)	Max depth (m)	Average depth (m)	Volume (10^6^ m^3^)	Secchi depth (m)	Mixing type	Trophic state
FAA (4)	46° 34′ N 13° 55′ E	Sep. 15 2015	555	2.20	29.5	16	35	4.2	holomictic- dimictic	oligotrophic
IRR (6)	47° 54′ N 13° 18′ E	Nov. 5 2015	553	3.60	32	15	53	7.1	holomictic-dimictic	oligo-mesotrophic
WEI (6)	46° 70′ N, 13° 38′ E	Sep. 14 2015	929	6.53	99	35	226	10	meromictic dimictic	oligotrophic
MON (12)	47° 49′ N 13° 22′ E	Sep 14 2016	481	13.80	68	36	497	7.8	holomictic-dimictic	mesotrophic
HAL (11)	47° 34′ N 13° 39′ E	Nov 17. 2015	508	8.60	125	65	558	9.5	holomictic monomictic	oligotrophic
MIL (10)	46° 48′ N 13° 35′ E	Sep. 16 2015	588	13.28	141	89	1205	10.1	meromictic monomictic	oligotrophic
TRA (17)	47° 52′ N 13° 48′ E	Nov. 24 2015	423	24.35	191	90	2189	8.8	holomictic monomictic^b^	oligotrophic
ATT (11)	47° 52′ N 13° 32′ E	Nov. 15 2015	469	46.20	171	84	3890	7.8	holomictic monomictic	(ultra)- oligotrophic

a Number of samples obtained from different depths are shown in parenthesis.

b at present meromictic.

### CARD-FISH and probe design

CARD-FISH of water samples was done following the protocol of Wendeberg (2010). In brief, water samples were fixed with 0.2 µm filtered formaldehyde (2% final concentration) and between 20 and 50 mL were filtered onto 0.2 µm white polycarbonate filters (Poretics, 47 mm filter diameter) and stored at −20°C until use. Before hybridization, filters were embedded in 0.1% ultrapure agarose (wt./vol., SeaKemVR LE Agarose, Lonza, Basel, Switzerland). Filters used for hybridization with bacterial oligonucleotide probes were incubated in lysozyme solution for 55 min at 37°C. Filters hybridized with archaeal oligonucleotide probes were pre-treated in lysozyme solution (see above), but with the addition of proteinase K (75 µL Proteinase K stock 1:100) for 35 min at 37°C. Taxonomic specificity, sequence information and hybridization conditions (formamide concentration) of HRP-labeled oligonucleotide probes (Biomers.net, Germany) are listed in Table S2 (Supporting Information). Alexa488 tyramides (Thermofisher) were used for signal amplification. The filters were counterstained with 4´,6-Diamidin-2-phenylindol (DAPI) and embedded in a 5:1:1 mix of Citifluor (Citifluor Ltd., London), Vectashield (Vector Laboratories, Inc., Burlingame, CA) and PBS. At least 400 DAPI stained cells were counted with a Zeiss Axioplan epifluorescence microscope.

Probe *Nitro878* was designed in ARB (Ludwig *et al*. 2004) using the SILVA database LSU Ref 123 (Pruesse *et al*. 2007). A bootstrapped maximum likelihood tree (GTR-GAMMA model) of all 23S rRNA sequences affiliated with *Nitrosomonas* (Fig. S3, Supporting Information) served as backbone for probe design with the ARB tools probe_design and probe_check. The resulting probe targets all publically available sequences affiliated with the target group (Fig. S3, Supporting Information), but has also nine outgroup hits within *Xanthomonadaceae* (Gammaproteobacteria). However, these microbes were obtained from non-aquatic habitats and can be thus neglected when using the probe for lake samples only. The probe and its competitor oligonucleotide were evaluated *in silico* with the web-tool mathFISH (Yilmaz, Parnerkar and Noguera 2011) and in the laboratory with a culture of *Nitrosomonas* sp. Is79 as positive control with different formamide concentrations to achieve stringent hybridization conditions.

### DNA-extraction and droplet digital PCR

Lake water samples (800 - 1170 mL) were filtered through polyethersulfone membrane filters (0.22 µm pore size and 47 mm diameter; Merck Millipore Ltd., Ireland) and stored at −20°C until use. DNA was extracted with the PowerWater©DNA Isolation Kit (MO BIO Laboratories Inc., USA) according to the manufacturer's protocol. A Quantus Fluorometer (Promega Corporation, USA) and QuantiFluor®dsDNA chemistry (Promega Corporation, USA) was used to measure the DNA content in the samples. Quantitative PCR analysis was performed using a QX200 Droplet Digital PCR system (Biorad) in combination with an automated droplet generator (AutoDG Instrument, Biorad). DdPCR reactions were set up with QX200 ddPCR EvaGreen Supermix (Biorad) to a final volume of 20 µL in 96-well plates following the manufacturer's instructions. Primer pairs used for qPCR targeting genes coding for key enzyme 4-hydroxybutyryl-CoA dehydratase in the HP/HB cycle were published in Alfreider *et al*. (2017). Primers targeting *aclA* genes of *Nitrospira* in the rTCA cycle and the putative *cbbL* form IA genes of the *N. oligotropha* cluster in the Calvin cycle were designed for this study (Table S1, Supporting Information). The optimal annealing temperature for both primer pairs was determined empirically with DNA extracted from samples of different lakes. Specification of all other primers used are also listed in Table S1 (Supporting Information). Optimal primer concentration and annealing temperature for most primers was (re)evaluated for ddPCR based on different primer concentrations and temperature gradient experiments. After automated droplet generation using the standard protocol provided by the manufacturer, PCR plates were heat sealed (Pierceable Foil Heat Seal, Biorad) and placed in a T100 thermal cycler (Biorad) for PCR amplification using the following cycling conditions: initial enzyme activation step of 5 min at 95°C, followed by 40 cycles including 30 s denaturation at 95°C, 30 s of primer annealing at primer specific temperatures (see Table S1, Supporting Information) and 1 min of primer extension at 72°C. Signal stabilization of the reaction was accomplished by final steps at 4°C for 5 min and 90°C for 5 min. A 2.5°C/sec ramp rate was used to guarantee each droplet reaches the correct temperature for each step. For signal measurement, the plates were placed into the reader and droplets were examined according to manufacturer's recommendations. Raw data were further analyzed using QuantaSoft Software 1.7.4. (Biorad). As the recommended dynamic range of the ddPCR system is from 1 to 120 000 copies of the target molecule/20 μL reaction, samples containing over 100 000 copies were diluted accordingly and analyzed again. Quality check included non-template controls, the evaluation of the fluorescence amplitude of positive and negative droplets and the examination of the reliability of the automated threshold settings by the QuantaSoft software.

### Sequence analysis for the evaluation of newly designed qPCR primers

In order to test the coverage and specificity of the newly designed qPCR primers pairs q_cbbL_IA_Nit and q_aclA_Nit. (Table S1, Supporting Information), sequence analysis of selected PCR-products from different lakes was performed (results are shown in Figs 4 and 5). All PCR products selected for sequencing analysis were separated on 1.5% agarose gels. Bands with proper size were selected for subsequent cloning, cut out of the gel and purified using a MinEluteVR Gel Extraction Kit (Qiagen Inc., Valencia, CA). PCR products were ligated into pGEM-T-Easy Vector plasmid (Promega, Madison, WI) and transformed into JM109 competent cells following the manufacturer's instructions. Clones were screened for the presence of proper inserts by PCR using vector-specific primers M13-F/R and GoTaqVR G2 Hot Start Master Mix (Promega, Madison, WI) following the protocol provided by the manufacturer. Selected reactions were Sanger sequenced by a sequencing service enterprise (Eurofins MWG Operon, Ebersberg, Germany).

Closest relatives to nucleotide sequences and deduced amino acid sequences were obtained using NCBI's sequence similarity search tools BLASTN and BLASTP (https://blast.ncbi.nlm.nih.gov/Blast.cgi). Deduced amino acids were aligned using MUSCLE algorithm as implemented in MEGA 6.0 software (Tamura *et al*. 2013), followed by visual inspection of the alignment. Neighbor-Joining trees applying gamma distribution as the distance method were also computed with the MEGA 6 software package. Bootstrap analysis (1000 replicates) was used to obtain confidence estimates for tree topology. The phylogenetic tree was condensed by compressing subtrees with highly similar sequences.

### Sequence data deposition

Sequences data have been submitted to GenBank databases under accession numbers MG600595 - MG600653 (*acl*) and MG600654 - MG600709 (*cbbL*-Form IA).

## RESULTS AND DISCUSSION

### Lake characteristics

**Figure 1.**
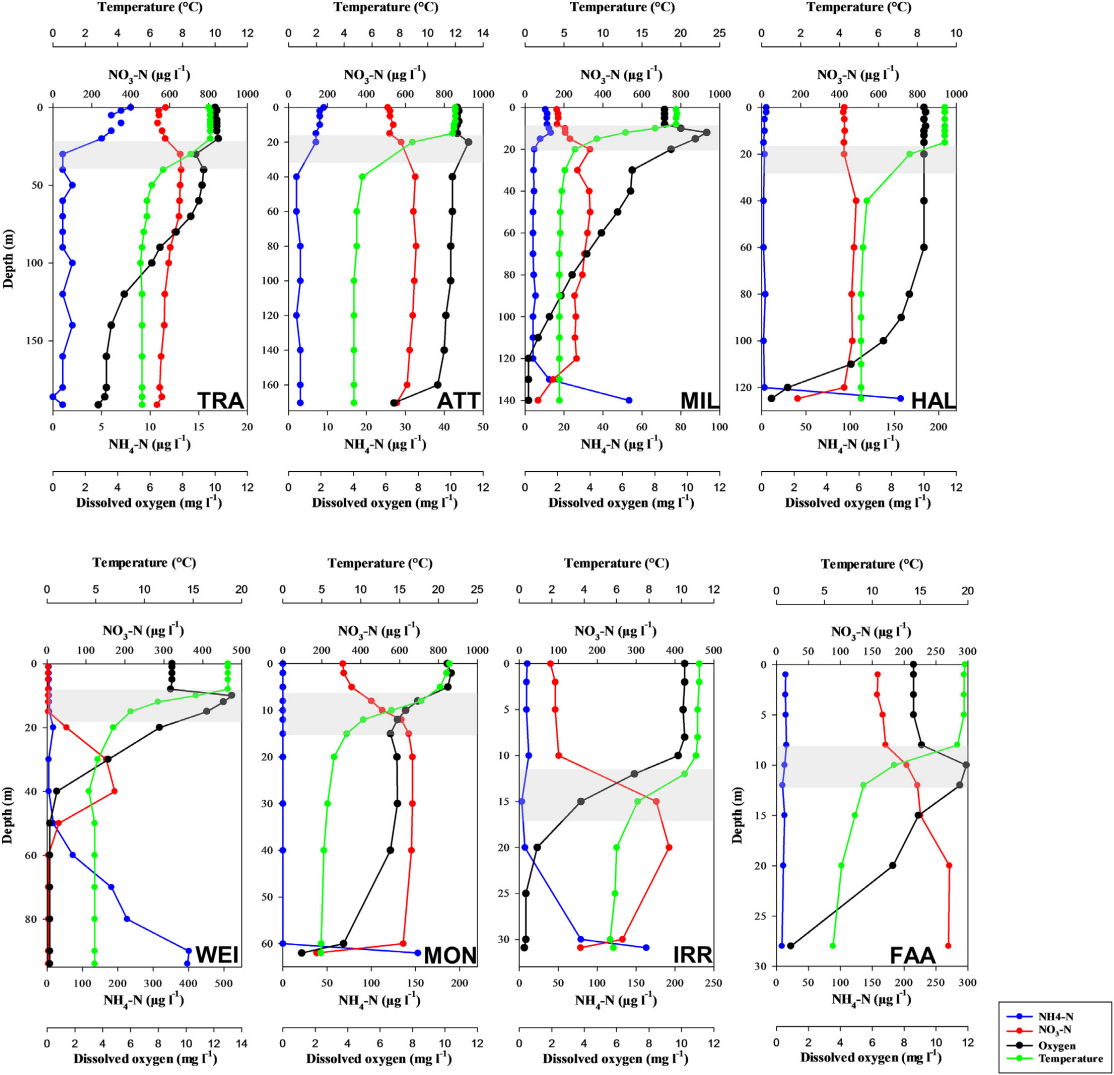
Water column profiles of ammonium, nitrate, temperature and DO in the lakes at the time of sampling. Please note the different scales between panels. The grey shaded area represents the approximate dimension of the metalimnion.

Vertical profiles of temperature revealed that at the time of sampling all lakes were stratified, with a thermocline established in different depths of each lake (Fig. 1). Deep lake ATT was well oxygenated over almost the entire water column, whereas all other lakes showed distinct declines in DO concentrations in the hypolimnion. Samples taken close to the bottom of the lakes FAA, HAL, IRR, MIL and WEI were characterized by DO concentration <1 mg L^−1^ (Fig. 1). Accordingly, most lakes revealed an increase in ammonium concentrations at the deepest sampling depths. Nitrate concentrations showed the highest values in the metalimnion, nitrate depletion caused by photoautotrophs occurred in the epilimnion of the lakes. In the hypolimnion, nitrate generally decreased with depth. A sharp decline in nitrate concentration was observed close to the lake bottom. Among the lakes, significant differences in hydrogencarbonate concentrations occur as this parameter is strongly influenced by the geology of the catchment (data not shown). However, hydrogencarbonate values range between 89 and 220 mg L^−1^, suggesting sufficient inorganic carbon concentrations for chemoautotrophs in all lakes. TRA exhibited high chloride concentrations in the hypolimnion (11.4 mg L^−1^ at 30 m increasing to and 49.3 mg L^−1^ at 191 m), caused by waste disposal of soda and salt industries.

### *Thaumarcheota* are the dominant nitrifiers in deep oligotrophic lakes

**Figure 2.**
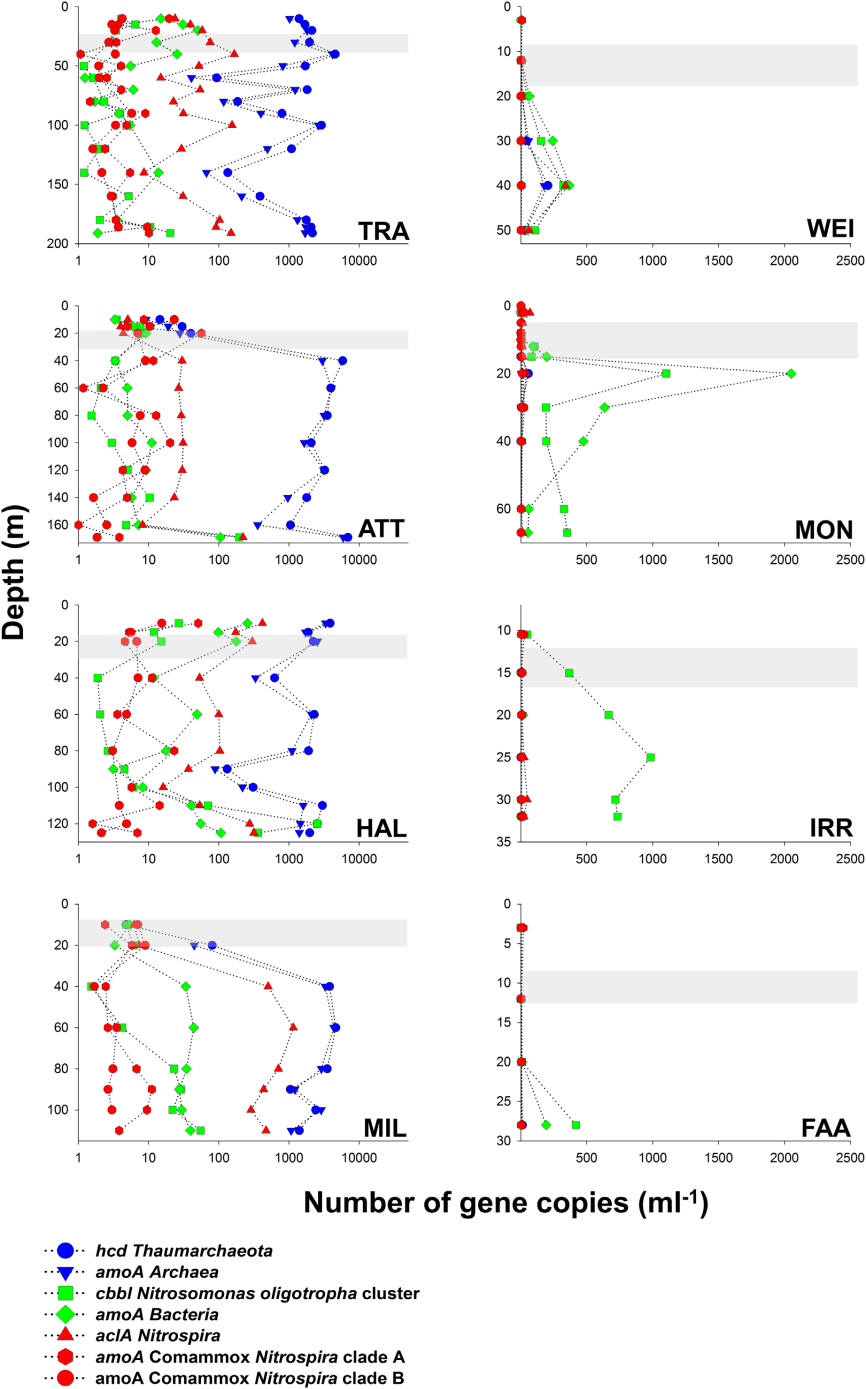
Vertical distribution of autotrophy-related (*hcd, cbbL andaclA*) and *amoA* gene copy numbers. Please note the logarithmic scale in the left side plots. The grey shaded area represents the approximate dimension of the metalimnion.

**Figure 3.**
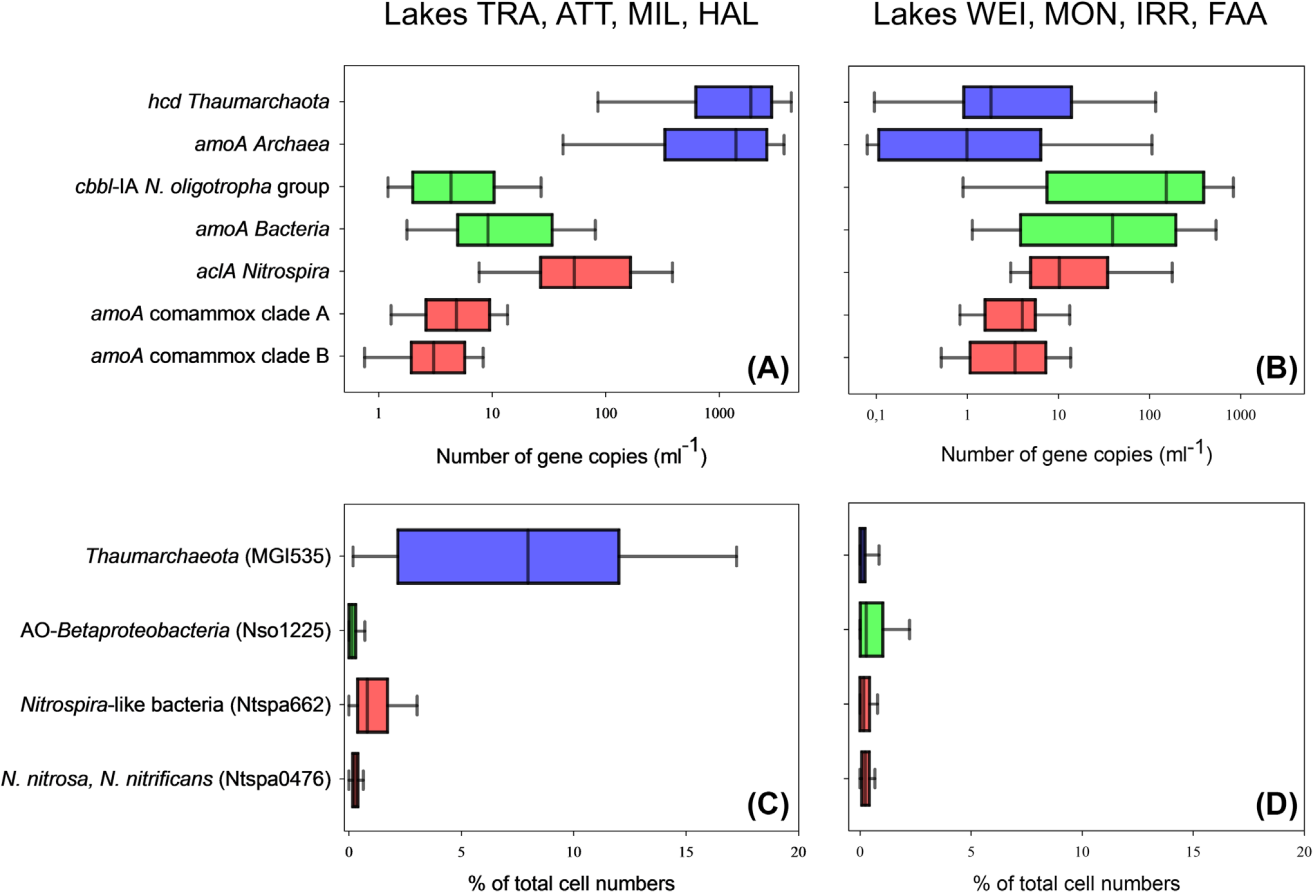
Box plots summarizing gene copy abundances (panels A and B) and CARD-FISH counts (panels C and D) in lakes dominated by AOA (panels A and C) and AOB (panels B and D). Note the logarithmic scale on the x-axis in panels A and B. Probe Ntspa0476 was originally designed to specifically detect Ca *N. nitrosa* and Ca *N. nitrificans* (van Kessel *et al*. 2015), but the probe also covers potential NOB. The vertical distribution of functional marker genes numbers and CARD- FISH counts are shown in detail in Fig. 2 and Fig. S1 (Supporting Information).

The quantification of *hcd* genes coding for the 4-hydroxybutyryl-CoA dehydratase in the HP/HB cycle indicates that *Thaumarchaeota* were the dominant nitrifiers in deep oligotrophic lakes (ATT, HAL, MIL and TRA). With exception of lakes HAL and TRA, the vertical distribution of *hcd* abundances was characterized by very low numbers in the upper water layers (Fig. 2). Thaumarchaeal *hcd* gene numbers in the hypolimnion were at least one magnitude higher than functional genes of bacterial nitrifiers (Figs 2 and 3). The maximum *hcd* abundance was observed in ATT at 170 m water depth (6.91 × 10^3^ genes mL^−1^). Smaller lakes (FAA, IRR, MON and WEI) were generally characterized by very low archaeal *hcd* gene numbers (<10 mL^−1^). The only exception was the hypolimnion of lake WEI, where *hcd* abundances reached values up to 0.21 × 10^3^ gene copies mL^−1^ (Fig. 2). Distribution patterns of archaeal *amoA* gene numbers were tightly correlated with *hcd* genes (r = 0,98; *P* < 0.001, Table 2) and on average almost equally abundant as *hcd* genes (Fig. 3). In accordance with the autotrophic gene marker, genes coding for archaeal AMO also showed the highest values in 170 m depth of ATT (5.93 × 10^3^ genes mL^−1^). Relative thaumarchaeal CARD-FISH counts (% of DAPI stained cells) were also significantly correlated with *hcd* (r = 0.67, *P* < 0.001) and *amoA* gene numbers (r = 0.66, *P* < 0.001, Table 2). With exception of the hypolimnion of lake MON, CARD-FISH numbers of *Thaumarchaeota* were close to the detection limit or absent in all smaller lakes (Fig. 3; Fig. S1, Supporting Information).

**Table 2.** Spearman rank correlation comparing different primers and probes used for the detection of specific guilds of nitrifiers. The taxonomic coverage is given in Tables S1 and S2 (Supporting Information).

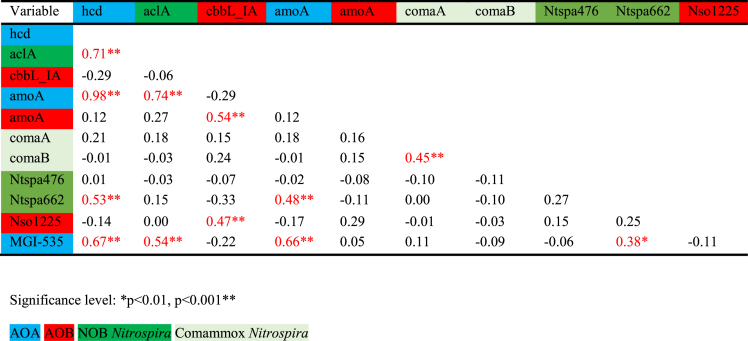

In freshwater systems, most research focusing on AOA are based on *amoA* gene surveys, suggesting that *Thaumarchaeota* are major players in the nitrogen cycle in deep lakes (e.g. Auguet *et al*. 2012; Vissers *et al*. 2013a,b; Hugoni *et al*. 2013). These results are also supported by studies based on rRNA analysis (including CARD-FISH) targeting *Thaumarchaeota* (Callieri *et al*. 2016). Callieri *et al*. (2014) amongst others already pointed out that the hypolimnion of deep oligotrophic lakes is also a place of important microbial metabolisms in regard to the carbon cycle and that the magnitude of dark CO_2_ fixation rates are comparable with photosynthetic fixation of inorganic carbon in the photic zone. Nevertheless, caution is required when linking thaumarchaeal abundances with potential archaeal chemoautotrophic activity, because several studies suggest that not all thaumarchaeal representatives are oxidizing ammonium and exhibiting heterotrophic or mixotrophic lifestyles (Herndl *et al*. 2005; Ingalls *et al*. 2006; Agogue *et al*. 2008; Alonso-Sáez *et al*. 2012). However, the highly similar distribution patterns of *amoA* and *hcd* gene numbers in this investigation provide strong evidence for a chemoautotrophic lifestyle of the thaumarchaeal communities at our study sites.

### *Nitrospira* observed in the lakes were mostly NOB, correlated with the distribution of AOA

**Figure 4.**
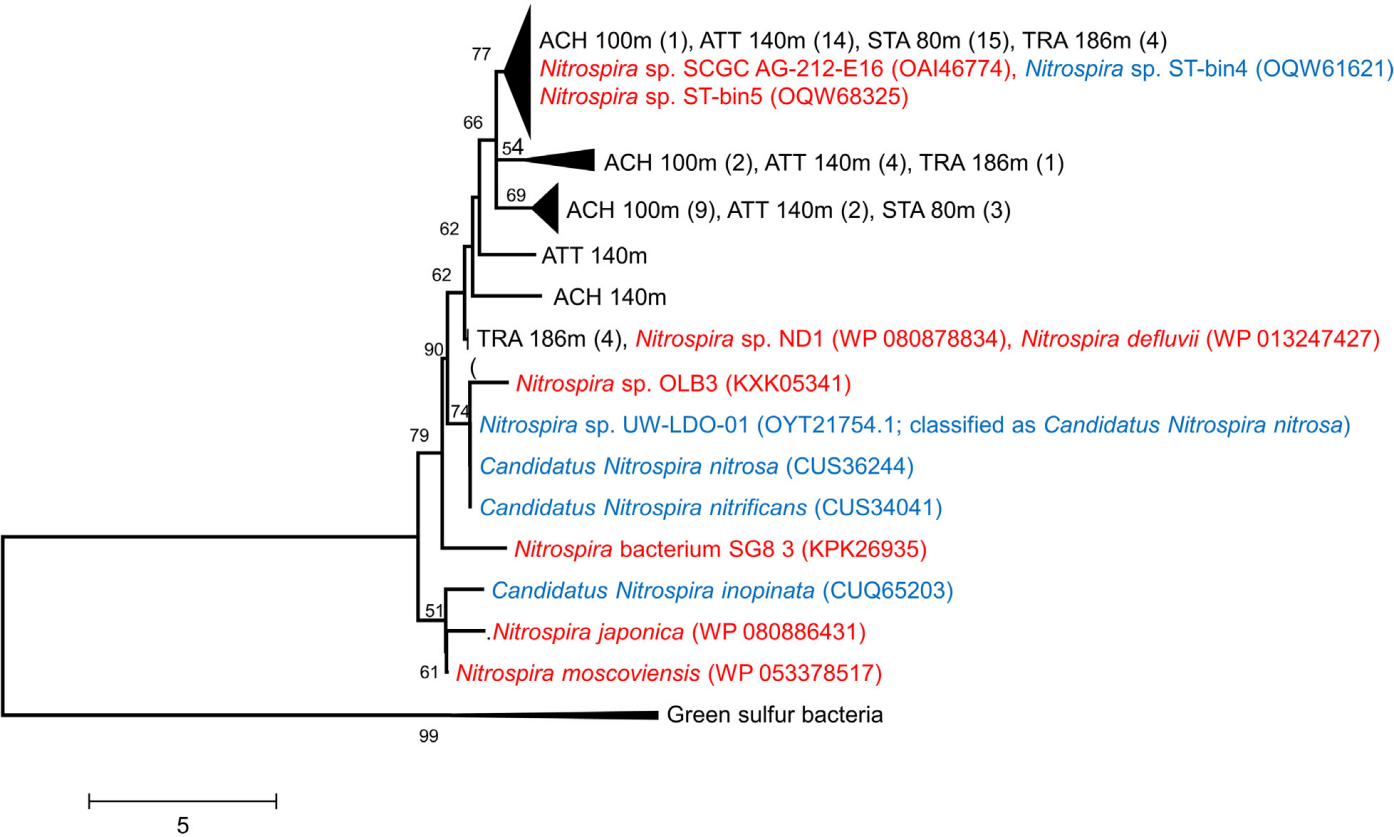
Evaluation of the specificity and coverage of qPCR primers targeting *aclA* genes in *Nitrospira* based on amino acid sequence analysis (shown in black). DNA was extracted from lake water samples obtained from this (ATT, TRA) and a previous study (Achensee-ACH, Starnberger See-STA; Alfreider *et al*. (2017)). Numbers in brackets indicate the number of clones analyzed. Closest NOB-*Nitrospira aclA* genes are shown in red color, comammox-*Nitrospira* are designated in blue.

Phylogenetic analysis of *aclA* genes, coding for the ATP-citrate lyase in the rTCA cycle, showed that *aclA* is not a suitable phylogenetic marker to differentiate comammox-*Nitrospira* from nitrite oxidizing *Nitrospira* (Fig. 4, Alfreider *et al*. 2017). The same is true for rRNA gene sequences, because comammox-*Nitrospira* do not form a monophyletic clade within *Nitrospira* (Pjevac *et al*. 2017). Consequently, our analysis based on CARD-FISH and *aclA* ddPCR quantified both, comammox and nitrite oxidizing *Nitrospira*, whereas *amoA*-targeted primers recently developed by Pjevac *et al*. (2017) allowed the specific quantification of comammox clades A and B. However, as both clades of comammox-*Nitrospira* were rare in all lakes (average *amoA* gene abundances <10 mL^−1^, Fig. 3), the majority of *Nitrospira* detected at our study sites are most probably related to NOB.

In accordance with thaumarchaeal *hcd* gene numbers, the highest abundances of *aclA* genes were also detected in the hypolimnion of the deep lakes, with the maximum number registered in MIL at 80 m depth (1.15 × 10^3^ genes mL^−1^, Fig. 2). In low AOA lakes, however, *Nitrospira* were also almost one magnitude less abundant (Fig. 3). Correlation analysis indicate that the distribution pattern of nitrite oxidizing *Nitrospira* based on *aclA* gene counts closely followed AOA (r = 0.74, *P* < 0.001; Table 2). This significant correlation in the distribution of AOA and NOB suggests biological interactions between both groups of nitrifiers. The co-occurrence of AOA and different guilds of NOB was also shown in other studies including freshwater ecosystems (Mukherjee *et al*. 2016) and a broad range of terrestrial habitats (Ke *et al*. 2013; Daebeler *et al*. 2014; Stempfhuber *et al*. 2015; Stempfhuber *et al*. 2017). However, the exact nature of this potential relationship is not completely elucidated, as microbe-microbe interactions were so far mostly studied between AOB and NOB (Daims, Lücker and Wagner 2016). The hypolimnion of deep lakes was also the environment where *Nitrospira*-like bacteria detected with CARD-FISH (probe Ntspa 662) were most abundant (Fig. 3), though the distribution patterns did not concur with the vertical gradients observed for *aclA* gene abundances (Table 2, Fig. 1 and Fig. S1, Supporting Information). With exception of the hypolimnion of WEI, *aclA* gene numbers were rare in smaller lakes (<100 *aclA* genes mL^−1^; Fig. 3).

### *Nitrosomonas-*related taxa are the major nitrifying group in smaller lakes

Primers specifically designed to target the CBB cycle for CO_2_ fixation in the *N. oligotropha* lineage revealed that these microbes were the most abundant nitrifiers in smaller lakes (Figs 2 and 3), with highest abundances of 1.1 × 10^3^ genes mL^−1^observed in MON in 20 m depth (Fig. 2). In large and deep lakes, numbers of *cbbL*-form IA genes were about two magnitudes lower than thaumarchaeal *hcd* abundances, however higher values were usually encountered in the deepest water layers (Figs 2 and 3). The maximal abundance was observed in ATT (6.9 × 10^3^ genes mL^−1^ in 170 m depth, Fig. 2). Abundances of AOB, quantified with a bacterial *amoA* primer set, were of similar magnitude with *cbbL* IA in both lake types (Fig. 3). The highest numbers were measured in MON in 20 m depth (2.05 × 10^3^ genes mL^−1^, Fig. 2). In contrast to archaeal marker genes for autotrophy and ammonia oxidation, *cbbL* IA and bacterial *amoA* genes showed a less pronounced correlation between and within the lakes (r = 0.54; *P* < 0.001, Table 2). Highest cell counts of AO-Betaproteobacteria (probe Nso1225) were usually observed in the deep water layers (Fig. S1, Supporting Information). CARD-FISH counts based on probe Nitro878, which was specifically designed to detect the *N. oligotropha* group, were mostly below the detection limit (data not shown). It is important to note, however, that other AOB might be present in the lakes that are not covered by both markers.

**Figure 5:**
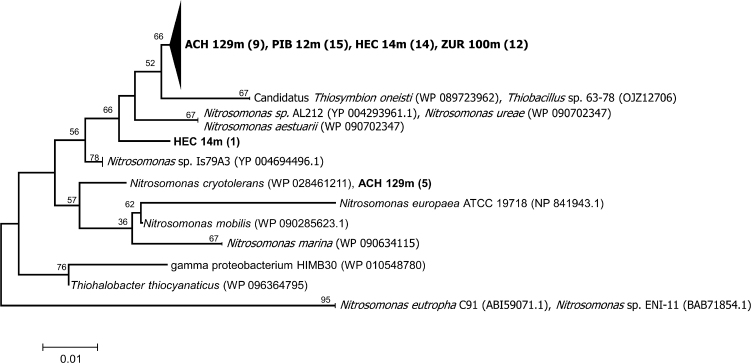
Phylogenetic tree reflecting the specificity and coverage of qPCR primers targeting *cbbL* genes in the *N. oligotropha* group based on amino acid sequence analysis derived in this study (shown in bold) and closest relatives obtained from GeneBank. Numbers in brackets indicate the number of clones analyzed. DNA extracts were obtained from lakes Achensee (ACH), Hechtsee (HEC), Piburger See (PIB) and Lake Zurich (ZUR), which were sampled in a former study (Alfreider *et al*. 2017).

One explanation for the discrepancy between different marker genes targeting AOB might be the taxonomic coverage and specificity of the primer sets used (Table S1, Supporting Information). Sequence analysis of PCR amplicons with the primer set q_cbbL_IA_Nit_f/q_cbbL_IA_Nit_r, which was specifically designed for the present study, showed that all form IA RubisCO sequences were affiliated with representatives of the targeted *N. oligotropha* lineage (*Nitrosomonas* cluster 6A) and closely related sister clades (Fig. 5). This group of AOB was found to be the dominating group of nitrifiers based on sequences derived by broad range primers for Form IA RubisCO genes in a variety of lakes (Alfreider *et al*. 2017). On the other hand, primers used for the quantification of *amoA* genes cover a wide range of proteobacterial AOB (Meinhardt *et al*. 2015), but primers do not perfectly match with the *cbbL* genes of several representatives of the *N. oligotropha* lineage (data not shown). Another reason might be the incongruent *cbbL* phylogeny of ammonia oxidizing Proteobacteria compared with rRNA based taxonomy, the latter gene was targeted by a CARD-FISH probe also designed to detect the *N. oligotropha* lineage (Table S2, Supporting Information). Although different forms of RubisCO are conserved proteins with distinct sequence differences, both horizontal gene transfer and gene duplication in proteobacterial lineages complicate the interpretation of systematic and physiological relationships based on RubisCO phylogeny (Delwiche and Palmer 1996; Tabita *et al*. 2008).

### Influence of environmental factors on distribution patterns

There is a fast growing number of studies investigating the environmental factors determining the distribution of different nitrifying guilds in nature. Particularly niche preferences of AOB and AOA have been extensively surveyed in the last years and several biotic and abiotic factors have been identified that determine their distribution in nature. So far, most investigations have been performed in soil and marine ecosystems, although patterns of niche differentiation of AOA and AOB were also studied in freshwater environments (Jiang *et al*. 2009; French *et al*. 2012; Small *et al*. 2013; Vissers *et al*. 2013a; Hayden and Beman 2014; Mukherjee *et al*. 2016; Pajares *et al*. 2017). In general, AOB dominantly contribute to nitrification under high substrate concentration while AOA are the most abundant group in oligotrophic systems.

**Figure 6.**
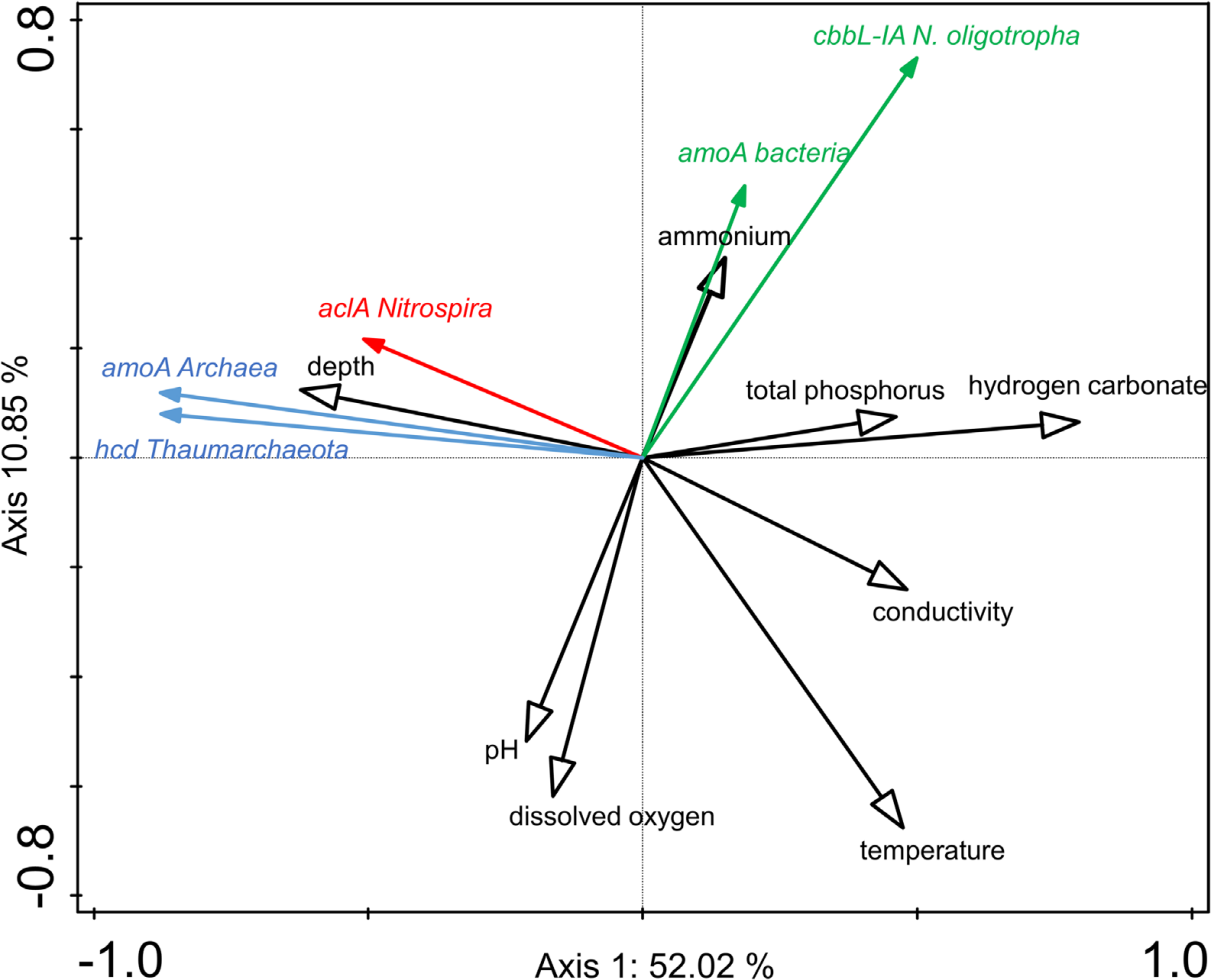
Redundancy analysis biplot of log gene copy numbers of different functional genes and selected environmental parameters obtained from different depths of eight lakes (n = 68). Copy numbers of both lineages of *amoA* genes in comammox-*Nitrospira* were not analyzed due to the low variability of the data close to the detection limit.

Potential niche preferences of AOA and AOB are usually not discussed from the perspective of different CO_2_ fixation strategies used by AOs, although different environmental conditions control their distribution in nature. The availability of ammonium and DO concentration were the determining factors for the occurrence of AOB at the study sites (Fig. 6). In deep lakes, at sampling depths where ammonium levels were very low, AOB were outnumbered by AOA by two or even three orders of magnitude (Fig. 2). The distribution of AOB was also positively correlated with the concentration of total phosphorus, suggesting that AOB and the high energy demand of the CBB cycle are better adapted to water depths characterized by an elevated nutrient status. Several studies have already reported that environments favor AOB development with higher substrate availability, including cultivation based investigations specifically targeting the *N. oligotropha* group (French *et al*. 2012). On the other hand, AOA using the most energy efficient aerobic carbon cycle (Könneke *et al*. 2014) were the dominant group in the hypolimnion of deep oligotrophic lakes and their distribution was positively correlated with depth (Fig. 6). Although not directly measured, depth dependent parameters such as competition for substrates with phototrophs, heavy grazing pressure on slow-growing nitrifiers and inhibition by light might be responsible for the low numbers of AOA in the surface waters of lakes (Hugoni *et al*. 2013; Small *et al*. 2013; Vissers *et al*. 2013a; Alfreider *et al*. 2017).

Several studies have revealed that AOA are better adapted to low oxygen concentrations than AOB (Lam *et al*. 2007; Martens-Habbena *et al*. 2009; French *et al*. 2012; Hugoni *et al*. 2013). These findings are not in accordance to our results, were DO was negatively correlated with *cbbL* gene numbers in AOB (Fig. 6). A low Km for O_2_ in AOA supports the hypothesis that AOA are well adapted to low O_2_ concentrations. On the other hand, the magnitude of DO is also of fundamental importance for the efficiency of the CBB cycle in organisms using this pathway (Berg 2011). Increased O_2_ levels in the catalytic environment of RubisCO enzymes in AOB have a negative effect on CO_2_ fixation abilities. In this context, one of the most important biochemical characteristics between different forms of RubisCO is the ability to discriminate between CO_2_ and O_2_ at a given CO_2_:O_2_ concentration ratio (Tabita 1999).

Although sequence analysis of different forms of RubisCO did not reveal a major cluster affiliated with NOB using the CBB cycle for CO_2_ fixation in lakes (Alfreider *et al*. 2017), we can for the moment only speculate that the genus *Nitrospira* is the most dominant NOB group at our study sites. In contrast to AOA and AOB, very little is known about the environmental factors determining the niche partitioning of NOB including *Nitrospira* in natural freshwater systems (Pester *et al*. 2014). Temperature, DO and nitrite levels have been shown to be key factors in niche differentiation of different NOB lineages (Daims, Lücker and Wagner 2016). At our study sites, multivariate statistical analysis showed that temperature is negatively correlated with the distribution of *Nitrospira aclA* gene abundances in the lakes, although different *Nitrospira* strains are known to grow in a broad temperature range (Alawi *et al*. 2009). It has been proposed that *Nitrospira* strains preferentially thrive under hypoxic conditions (Park and Noguera 2008) due to the lack of reactive oxygen species as a classic defense mechanism against oxidative stress (Lücker *et al*. 2010). Our results demonstrated the presence of *Nitrospira*-like bacteria (which also includes potential comammox-*Nitrospira)* in both high and low DO habitats and statistical analysis suggests that oxygen concentrations had no crucial effect on the distribution of *Nitrospira*-like bacteria in the lakes (Fig. 6).

Redundancy analysis of the relationship between environmental parameters and CARD-FISH counts showed a similar trend observed with the ddPCR results of AOA and NOB based on functional genes (Fig. S2, Supporting Information). However, in contrast to the RDA analysis based on *amoA* and *cbbL* abundances, the availability of ammonium and DO concentration were not the controlling variables for the distribution of AOB in lakes based on CARD-FISH numbers. One explanation for this disagreement might be taxonomic coverage and specificity of the different markers used, which was already discussed above. Consequently, it can also not be ruled out that some representatives detected by CARD-FISH have a heterotrophic lifestyle. Another reason for discrepancy are the different detection limits of the two approaches (Alfreider *et al*. 2017). Gene copy numbers analyzed by ddPCR were measured as low as one target gene mL^−1^ in the lake water samples, while microscopic-based techniques do not allow accurate counts at this low magnitude. As AOB mostly occur in very low abundances at the study sites, the efficiency of both methods might cause corresponding differences in the results.

One major question, however, remains unclear: Why is the third group of AOs, comammox-*Nitrospira*, so rare in the studied lakes? This stands in contrast to a recent investigation suggesting that the comammox bacterium *N. inopinata* is highly adapted to oligotrophic habitats, at least based on results derived from substrate competition kinetics (Kits *et al*. 2017). Studies that investigate the influence of environmental variables on the structure or distribution of comammox are rare. Beside the proposed adaptation of comammox-*Nitrospira* to microaerophilic and low substrate fluxes (Lawson and Lücker 2018), Fowler *et al*. (2018) identified temperature to have a positive impact on *Nitrospira* in rapid sand filters. However, the authors could not distinguish if comammox and nitrite-oxidizing taxa were affected. In principle, the high adaptation of comammox-*Nitrospira* to oligotrophic conditions is also reflected by the rTCA cycle, a pathway that is considered to be far more energy efficient than other CO_2_ fixation cycles employing non-reducing carboxylases (Berg 2011; Mangiapia and Scott 2016). However, some taxa including the thermophilic *Hydrogenobacter thermophiles* and the genus *Nitrospira*, have developed enzymatic adaptations for oxygen tolerance, in contrast to most other bacterial phyla operating this cycle in anaerobic or microaerobic environments (Yamamoto *et al*. 2006; Lücker *et al*. 2010; Berg 2011; Daims *et al*. 2015). The specific biochemical adaptations are poorly understood and some still unknown mechanisms may strengthen the O_2_ robustness of the rTCA pathway in *Nitrospira* (Berg 2011). However, if these protection mechanisms are accompanied by a lower specific activity, it may significantly increase the energy requirements of the pathway when used in an oxic environment (Berg 2011). Certainly, more research is necessary to determine the niche preferences of comammox-*Nitrospira*, focusing on habitats characterized by different trophic states and oxygen levels.

## Supplementary Material

Supplementary Data
