# Supplementary material for: Autotrophic carbon fixation strategies used by nitrifying prokaryotes in freshwater lakes
Source: FEMS Microbiol Ecol. 2018 Aug 18;94(10):fiy163. doi: 10.1093/femsec/fiy163 (PMC6118323; doi:10.1093/femsec/fiy163)
Supplement: Supplementary Data [file fiy163_supplemental_tables_and_figures.docx]

**Supplementary Information**

Autotrophic carbon fixation strategies used by nitrifying prokaryotes in freshwater lakes

A Alfreider, V Grimus, M Luger, A Ekblad, MM Salcher and M Summerer

**Supplementary Tables**

Table S1: Specification of primers used for qPCR

Table S2: Specification of probes used for CARD-FISH

**Supplementary Figures**

Figure S1: Vertical profiles of CARD-Fish counts

Figure S2: RDA of CARD-FISH counts and environmental parameters

Figure S3: Coverage of CARD-FISH probe Nitro878

**Supplementary Table S1** Specification of primers used for ddPCR used in this study

Function/Targeted protein Coverage Primer Name Sequence (5´-3´) Ta^1^ Pconc^2^ Reference

**Ammonia oxidizers**

RubisCO form IA *N. oligotropha* cluster q_cbbL_IA_Nit_f GGYATTCARGTWGAACGYGATCG 60 0.25 This study

(CBB cycle)´ q_cbbL_IA_Nit_r TTGGCAAGCYTCYACCACGAATT

4-hydroxybutyryl- CoA dehydratase *Thaumarchaeota* 1.1a qPCR_hcd_f GACTGATCCWAAAGGDGAYAGAAG 56 0.25 Alfreider et al. 2017

(HP/HB cycle) qPCR_hcd_r CCYTTARCATCTGCWGGAATTGC

Ammonia monooxygenase AOA GenAOAF ATAGAGCCTCAAGTAGGAAAGTTCTA 55 0.1 Meinhardt et al., 2015

GenAOAR CCAAGCGGCCATCCAGCTGTATGTCC

Ammonia monooxygenase AOB amoA-1Fmod CTGGGGTTTCTACTGGTGGTC 58 0.1 Meinhardt et al., 2015

GenAOBR GCAGTGATCATCCAGTTGCG

**Comammox and nitrite oxidizers**

ATP citrate lyase (rTCA cycle) *Nitrospira* q_aclA_Nit-f TCSTTCGGCGTCATYACCAAG 56 0.25 This study

q_aclA_Nit-r GCCSGCATGRCCGAAYTTCAT

**Comammox**

Ammonia monooxygenase Comammox *Nitrospira* clade A comaA-244f_a ACAACTGGGTGAACTA 52 0.25 Pjevac et al., 2017

comaA-244f_b TATAACTGGGTGAACTA

comaA-244f_c TACAATTGGGTGAACTA

comaA-244f_d TACAACTGGGTCAACTA

comaA-244f_e TACAACTGGGTCAATTA

comaA-244f_f TATAACTGGGTCAATTA

comaA-659r_a AGATCATGGTGCTATG

comaA-659r_b AAATCATGGTGCTATG

comaA-659r_c AGATCATGGTGCTGTG

comaA-659r_d AAATCATGGTGCTGTG

comaA-659r_e AGATCATCGTGCTGTG

comaA-659r_f AAATCATCGTGCTGTG equimolar primer mixtures

Ammonia monooxygenase Comammox Nitrospira clade B comaB-244f_a TAYTTCTGGACGTTCTA 52 0.25 Pjevac et al., 2017

comaB-244f_b TAYTTCTGGACATTCTA

comaB-244f_c TACTTCTGGACTTTCTA

comaB-244f_d TAYTTCTGGACGTTTTA

comaB-244f_e TAYTTCTGGACATTTTA

comaB-244f_f TACTTCTGGACCTTCTA

comaB-659r_a ARATCCAGACGGTGTG

comaB-659r_b ARATCCAAACGGTGTG

comaB-659r_c ARATCCAGACAGTGTG

comaB-659r_d ARATCCAAACAGTGTG

comaB-659r_e AGATCCAGACTGTGTG

comaB-659r_f AGATCCAAACAGTGTG equimolar primer mixtures

^1^ Ta: Annealing temperature (°C); ^2^ Primer concentration (µmol)

**Supplementary Table S2** Specification of CARD-FISH probes used in this study

Function/Target rRNA Coverage Probe name Sequence (5´-3´) Formamide (%)^1^ Reference

**Ammonia oxidizers**

16S rRNA *Thaumarchaeota* Marine group I MGI 535 TCC TGA CCA CTT GA GG TGC TGG 45 Coci *et al*., 2015

16S rRNA mostly *Nitrosomonas, Nitrosospira* Nso1225 CGC CAT TGT ATT ACG TGT GA 35 Mobarry et al. 1996

23S rRNA *Nitrosomonas oligotropha* cluster Nitro878 AAG TCT CAA TGA CCC CCT 20 This study

23S rRNA Competitor for Nitro878 cNitro878 AAG TCG CGA TGA CCC CCT 20 This study

**Comammox and nitrite oxidizers**

16S rRNA *Nitrospira* Ntspa662 GGA ATT CCG CGC TCC TCT 35 Daims *et al.,* 2001

16S rRNA Competitor for Ntspa662 cNtspa662 GGA ATT CCG CTC TCC TCT 35 Daims *et al.,* 2001

16S rRNA Ca. *N. nitrosa*, Ca. *N. nitrificans*^1^ Ntspa0476 CTG CAG GTA CCG TCC GAA 20 van Kessel *et al*., 2015

16S rRNA Competitor for Ntspa0476 cNtspa0476 CTG GAG GTA CCG TCC GAA 20 van Kessel et al., 2015

^1^ rRNA based analysis does not allow to distinguish between comammox or strict NOB Nitrospira (Pjevac *et al.,* 2017)

**Supplemental Figure S1** Vertical distribution of different nitrifiying guilds based on CARD-FISH counts (% of DAPI stained cells)

Figure S2. Redundancy analysis biplot of CARD-FISH counts and selected environmental parameters obtained from different depths of eight lakes (n=56).

**Supplemental Figure S3** Bootstrapped maximum likelihood tree of 23S rRNA genes targeted by probe Nitro878 and their closest relatives. Labels in grey indicate partial sequences.

**
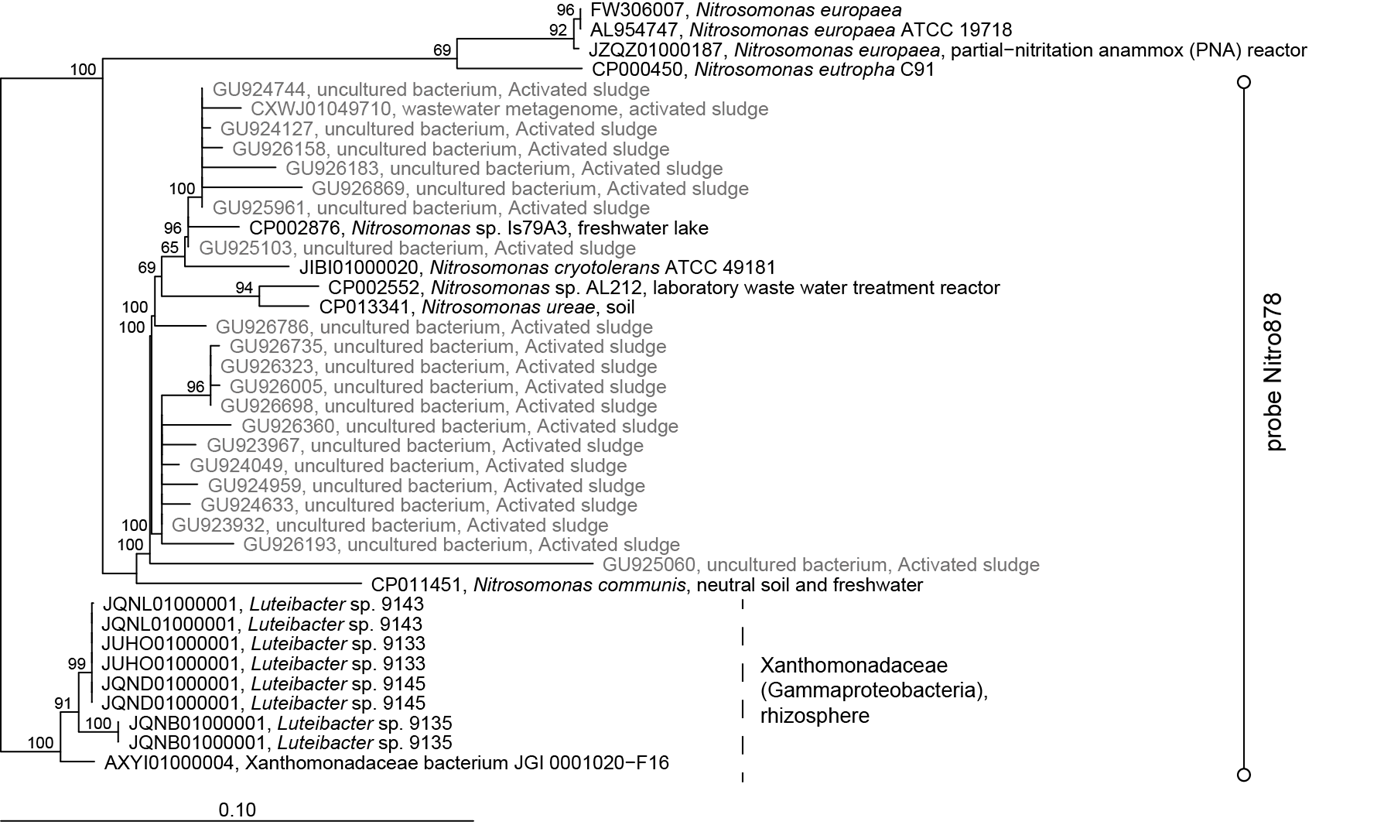
**
